# Solitary Fibrous Tumor Requiring Abdominal Aortic Vessel Replacement

**DOI:** 10.7759/cureus.68251

**Published:** 2024-08-30

**Authors:** Naoto Tanabe, Satoshi Tokuda, Erina Nagai, Hideyuki Katayama, Hideyuki Kanemoto

**Affiliations:** 1 Department of Gastroenterological Surgery, Shizuoka General Hospital, Shizuoka, JPN; 2 Department of Cardiovascular Surgery, Shizuoka General Hospital, Shizuoka, JPN

**Keywords:** tumor remnant, artificial blood vessel replacement, surgical treatment, retroperitoneal tumor, solitary fibrous tumor (sft)

## Abstract

Solitary fibrous tumors (SFTs) are mesenchymal tumors, and retroperitoneal occurrence is rare. It has been identified in a variety of soft tissues and organs, such as the pleura, peritoneum, and meninges. In this case, the tumor was in contact with the abdominal aorta, and the invasion was difficult to judge preoperatively. Intraoperatively, it was revealed that the tumor could not be completely removed without aortic replacement.

Although SFTs have a generally good prognosis, certain factors, such as tumor incomplete resection, have been reported to increase the risk of recurrence and metastasis. We were able to completely remove the tumor by performing a combined resection of the aorta. The specimens were microscopically disorganized proliferation of spindle-shaped cells. Immunostaining was positive for cluster of differentiation 34 (CD34) and signal transducer and activator of transcription 6 (STAT6). The tumor cells infiltrating into aortic adventitia were observed. This is a valuable case in which artificial blood vessel replacement was able to reduce the risk of recurrence and metastasis due to tumor remnants. We report a rare case of SFT resected with artificial blood vessel replacement.

## Introduction

Solitary fibrous tumors (SFTs) were first described by Klemperer and Coleman [1] in 1992 as malignant neoplasms that arise in the pleura.

SFTs are composed of various pleomorphic spindle-shaped cells mixed with collagen fibers. They are characterized by mixed collagen, arranged in disorganized, short fascicles.

SFTs develop primarily within the thoracic cavity; however, they can occur in various anatomic sites, with approximately 30%-40% of SFTs detected outside the thoracic cavity and only 1% detected in the retroperitoneum [2]. Ten percent to 15% of SFTs are malignant, and those with higher mitotic counts, necrosis, or calcification on pathological examination represent aggressive neoplasms.

Surgical resection is the standard of care, while there are no established guidelines on chemotherapy or radiation therapy [3]. However, the incidence of local recurrence or distant metastasis after surgical resection depends on many factors such as the patient’s age, initial tumor diameter, the presence of residual tumor, and pathological findings indicating highly aggressive malignancy [4].

In this report, we describe a case of SFT in the retroperitoneum that required an artificial vascular replacement of the abdominal aorta. This article was previously presented as a meeting abstract at the 85th Annual Congress of Japan Surgical Association on November 16-18, 2023.

## Case presentation

A 42-year-old male presented an abdominal mass detected on ultrasonography during a medical check-up.

**Figure 1:**
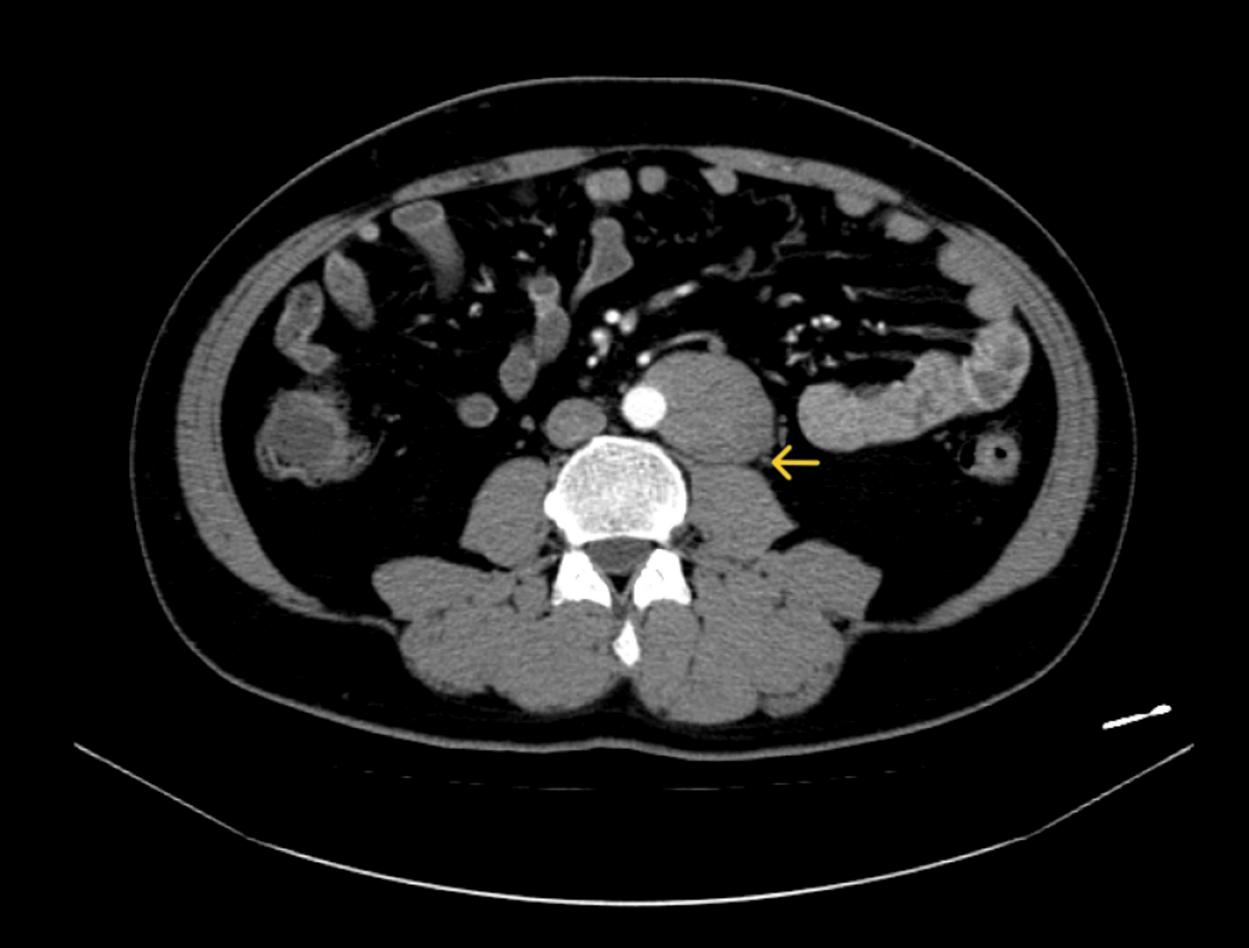
Preoperative contrast CT scan A 65 mm-long mass semi-peripheral to the aorta is seen on the left side of the aorta. There is no intervening fat layer between the aortic wall and the tumor. The tumor is in contact with the left ureter (arrow) CT: computed tomography

**Figure 2:**
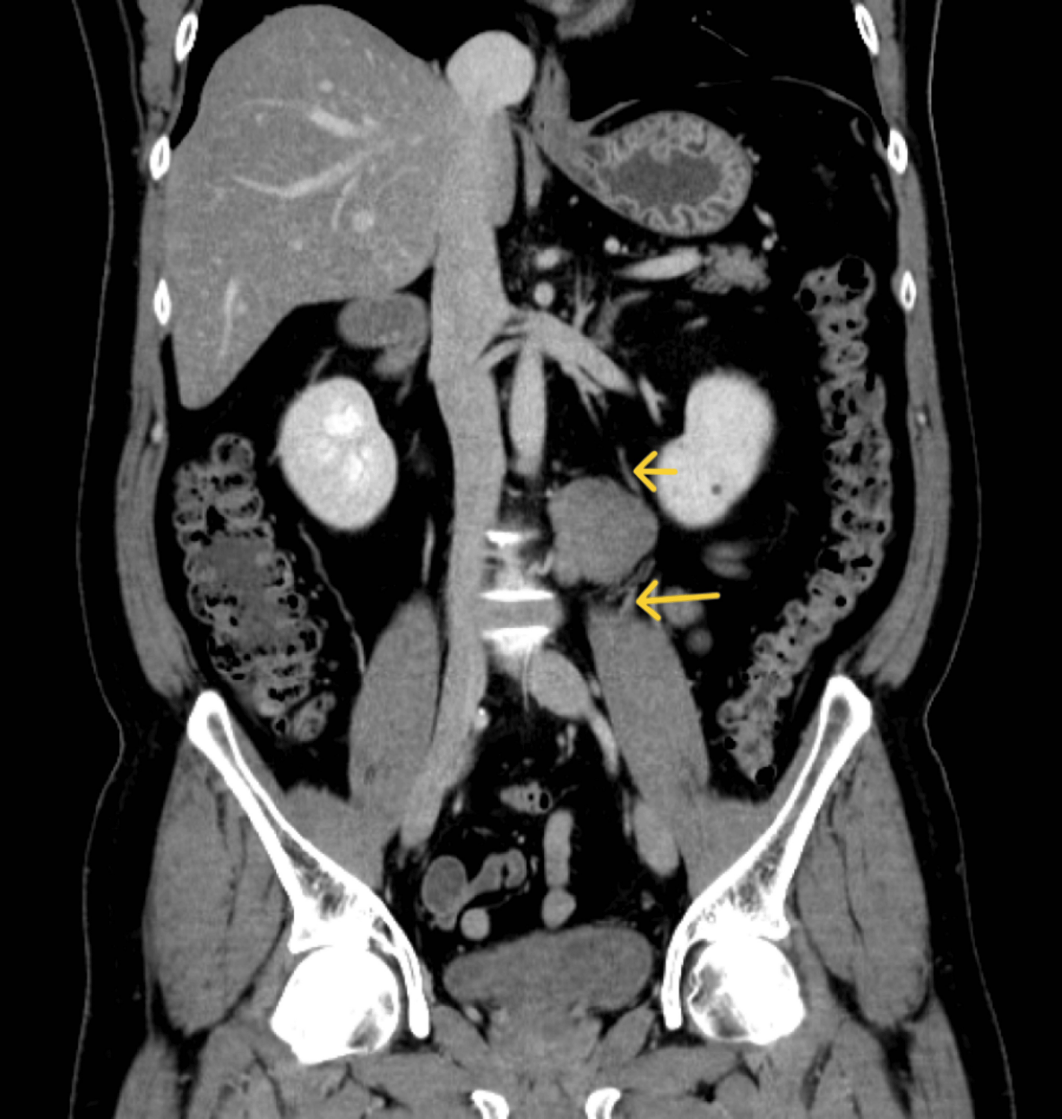
Preoperative contrast CT scan The tumor is in contact with the left ureter (arrows) CT: computed tomography

**Figure 3:**
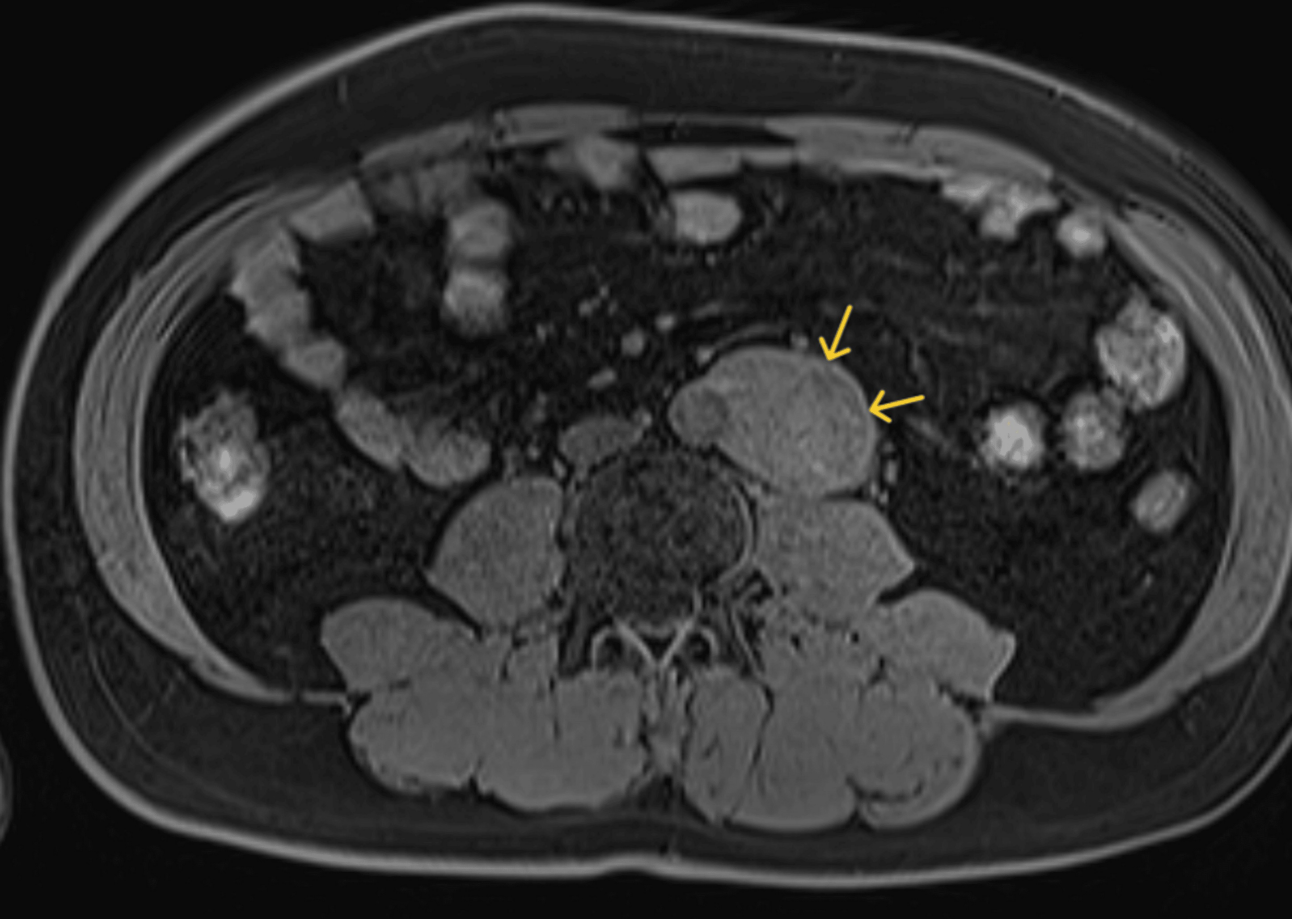
MRI T1-enhanced image The solitary fibrous tumor (arrows) was seen tangential to the left side of the abdominal aorta, and there was no interposition of fatty layer between the abdominal aorta and the SFT MRI, magnetic resonance imaging; SFT, solitary fibrous tumor

Abdominal computed tomography (CT) and magnetic resonance imaging revealed a tumor measuring approximately 65 mm tangential, surrounding the left side of the abdominal aorta, without the interposition of the fatty layer (Figures 1-3).

**Figure 4:**
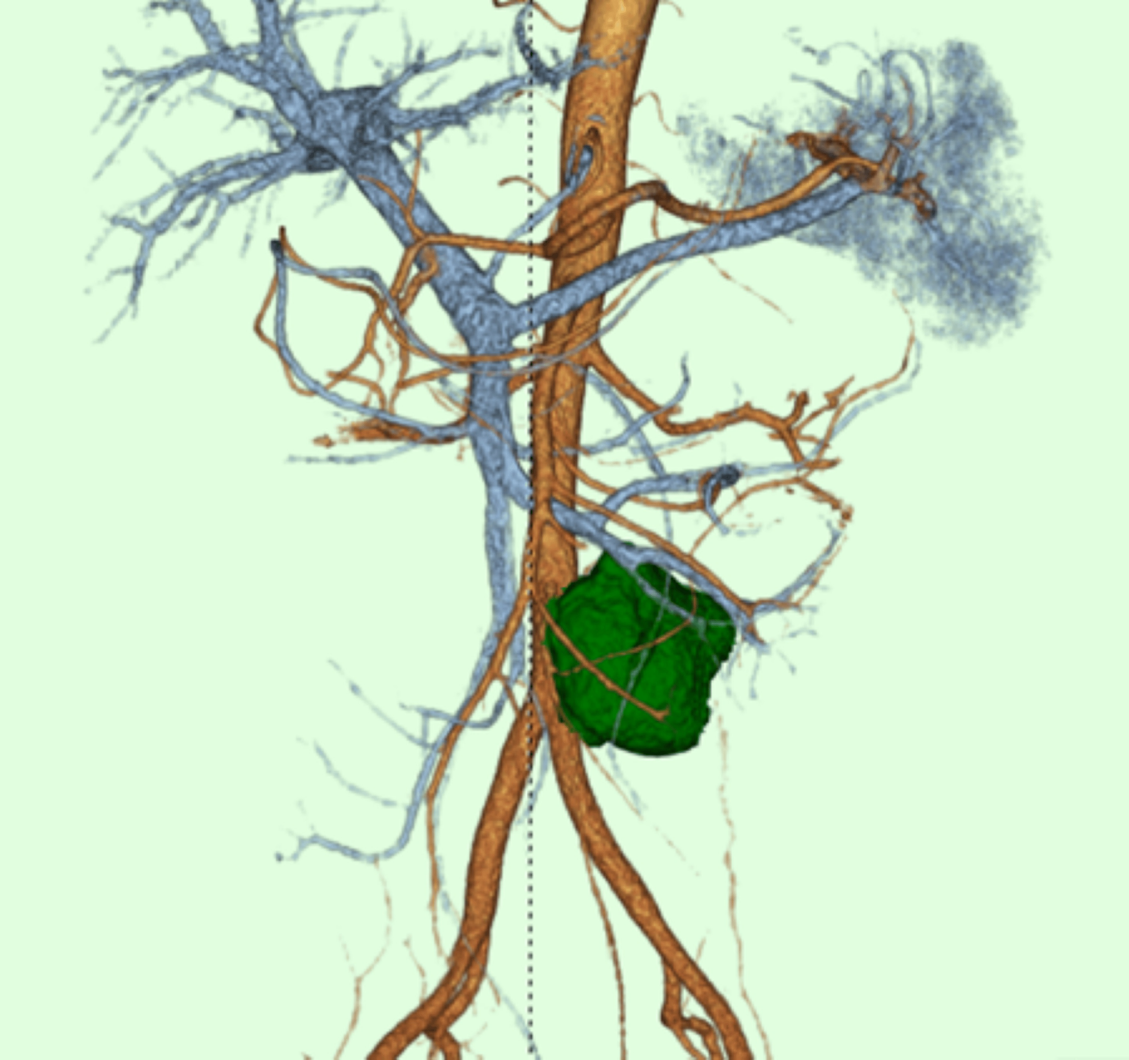
3D CT The tumor is near the inferior mesenteric artery and the inferior mesenteric vein CT: computed tomography

The tumor was located near the inferior mesenteric artery and the inferior mesenteric vein (Figure 4).

**Figure 5:**
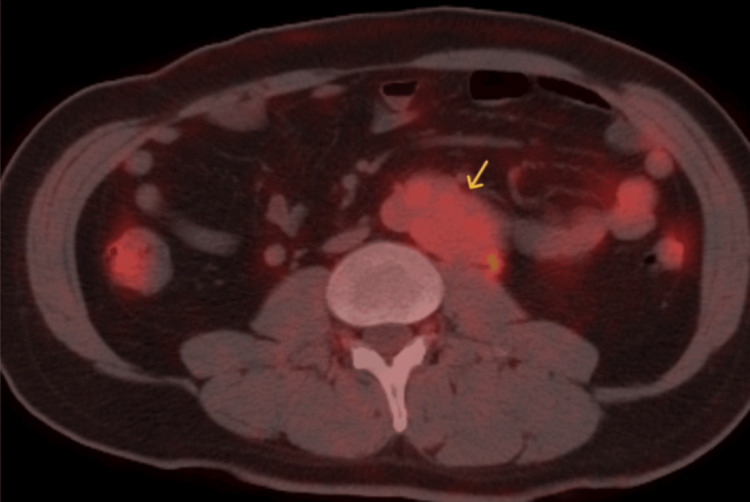
PET-CT scan There is a low-intensity tumor (arrow) with an SUVmax of approximately 2.5, with no discernible signs suggestive of distant metastasis. The tumor is in contact with the left ureter PET, positron emission tomography; CT, computed tomography; SUVmax, maximum standardized uptake value

Positron emission tomography-CT revealed a low fludeoxyglucose F18 uptake tumor with a maximum standardized uptake value (SUVmax) of approximately 2.5 and no signs of distant metastasis (Figure 5).

**Figure 6:**
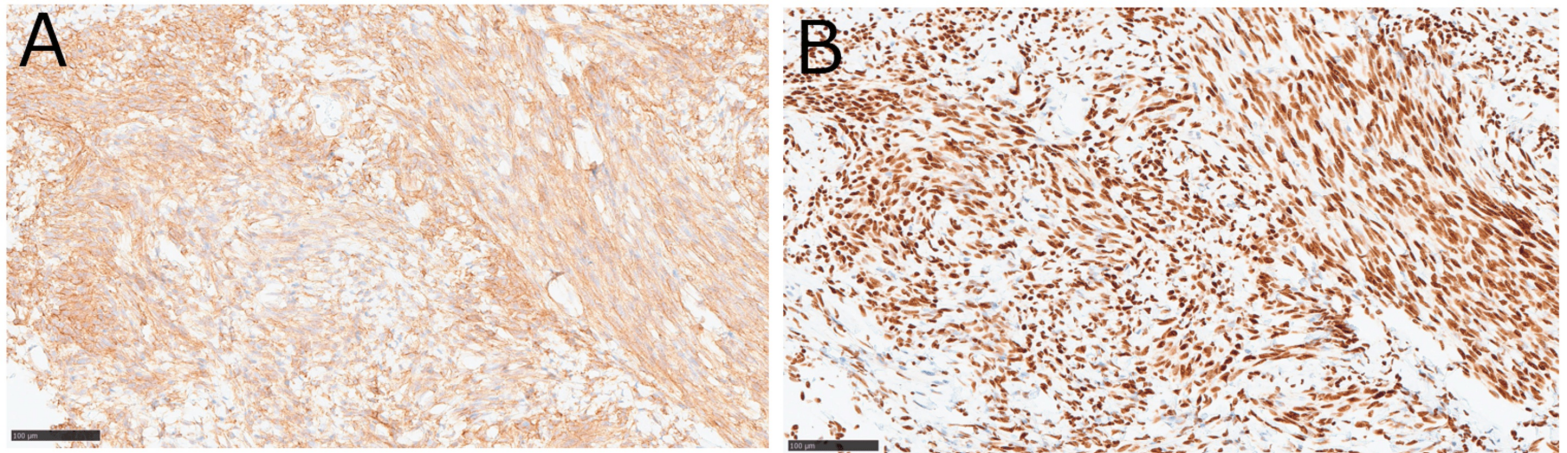
Immunohistochemical staining Immunohistochemical staining was positive for CD34 (A), as well as signal transducer and activator of transcription 6 (B). The magnification for both is ×100 CD34: cluster of differentiation 34

CT-guided biopsy was performed, and pathological examination revealed spindle cell proliferation with mild-to-moderate cellular atypia. Immunostaining was positive for cluster of differentiation 34 (CD34) and signal transducer and activator of transcription 6 (STAT6), and the Ki-67 index was <5% (Figure 6).

The preoperative diagnosis was SFT. On imaging, the tumor was in contact with the left abdominal aorta; however, no obvious invasion was noted, and the tumor was considered dissectible. A surgical resection was scheduled.

Surgical procedure

**Figure 7:**
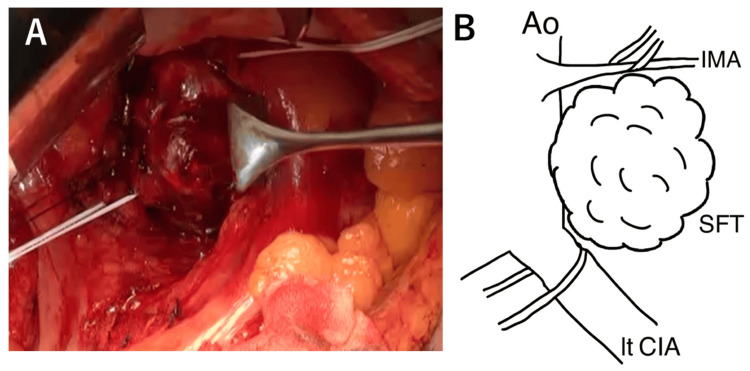
Surgical image (A) and schema (B) The tumor in the retroperitoneal cavity is detached from the surrounding stromal tissue, and the inferior mesenteric artery and left common iliac artery are taped. Schema credits: Naoto Tanabe IMA, inferior mesenteric artery; SFT, solitary fibrous tumor; CIA, common iliac artery; Ao, aorta

The operation was performed via laparotomy. Owing to the proximity of the tumor to the left ureter, a left ureteral stent was placed prior to laparotomy. The prehypogastric nerve fascia was incised to reach the posterior rectal space. The left common iliac artery was encircled. A cephalic tumor was identified in the area near that site (Figure 7).

**Figure 8:**
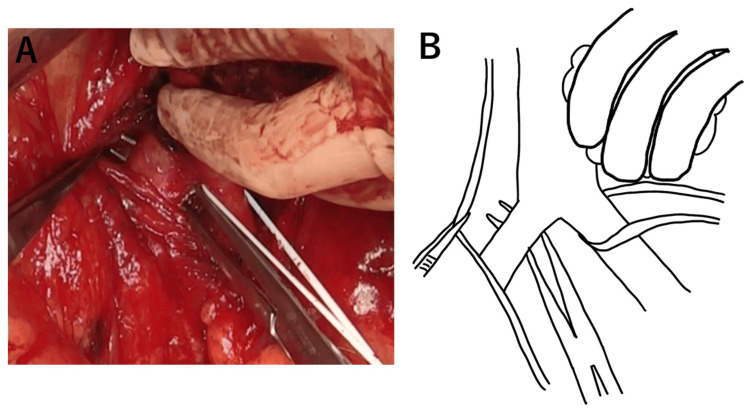
Surgical image (A) and schema (B) The aortic wall and some areas were difficult to dissect; aortic complications required resection and the taping of major vessels. Bilateral common iliac arteries were taped. Schema credits: Naoto Tanabe

The inferior mesenteric artery was taped and dissected around the tumor. The tumor and abdominal aorta were bluntly and sharply dissected. Bleeding from the abdominal aorta near the center of the tumor was observed, and it was controlled as much as possible by compression. The abdominal aorta and the right common iliac artery above the tumor level were encircled (Figure 8).

**Figure 9:**
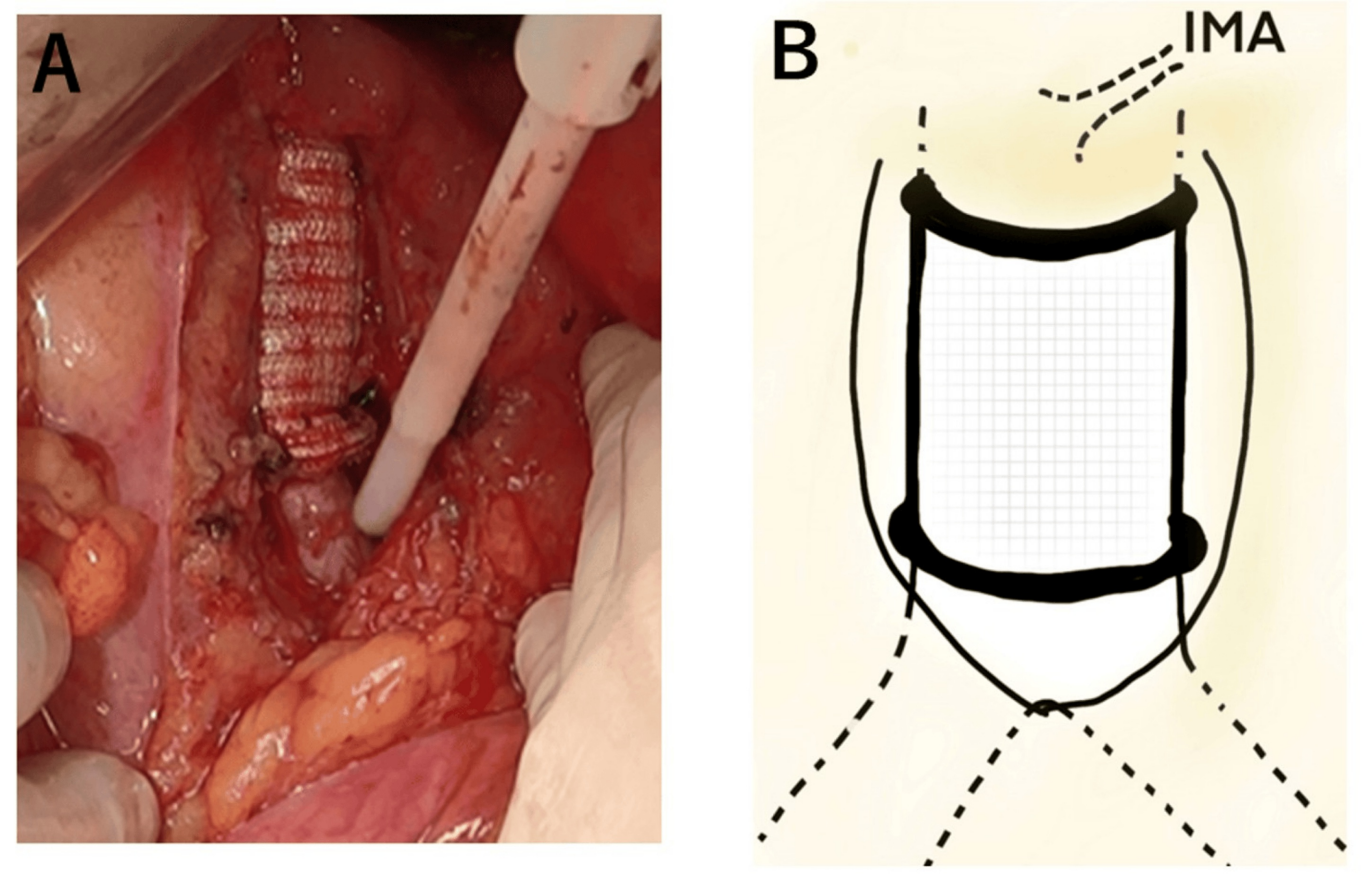
Surgical image (A) and schema (B) Hemorrhage occurred as we proceeded to dissect the tumor from the aortic wall. First, most of the tumor was resected to secure the visual field. The remnant tumor on the adventitia and a part of the aorta was resected in combination after clamping the abdominal aorta and right and left common iliac arteries, and a type I artificial vessel replacement was performed just below the inferior mesenteric artery. Schema credits: Naoto Tanabe IMA: inferior mesenteric artery

The biggest part of the tumor was resected, and the view around the abdominal aorta was cleared. After clamping the abdominal aorta and right and left common iliac arteries, the remnant tumor on the aortic adventitia was completely removed by external resection involving the aorta. Thereafter, a type I artificial vessel replacement was performed just below the inferior mesenteric artery (Figure 9).

A drainage tube was placed in the retroperitoneal space to quantify bleeding. The operative time was three hours and 39 minutes, and the blood loss was 1,080 mL.

Pathological findings

**Figure 10:**
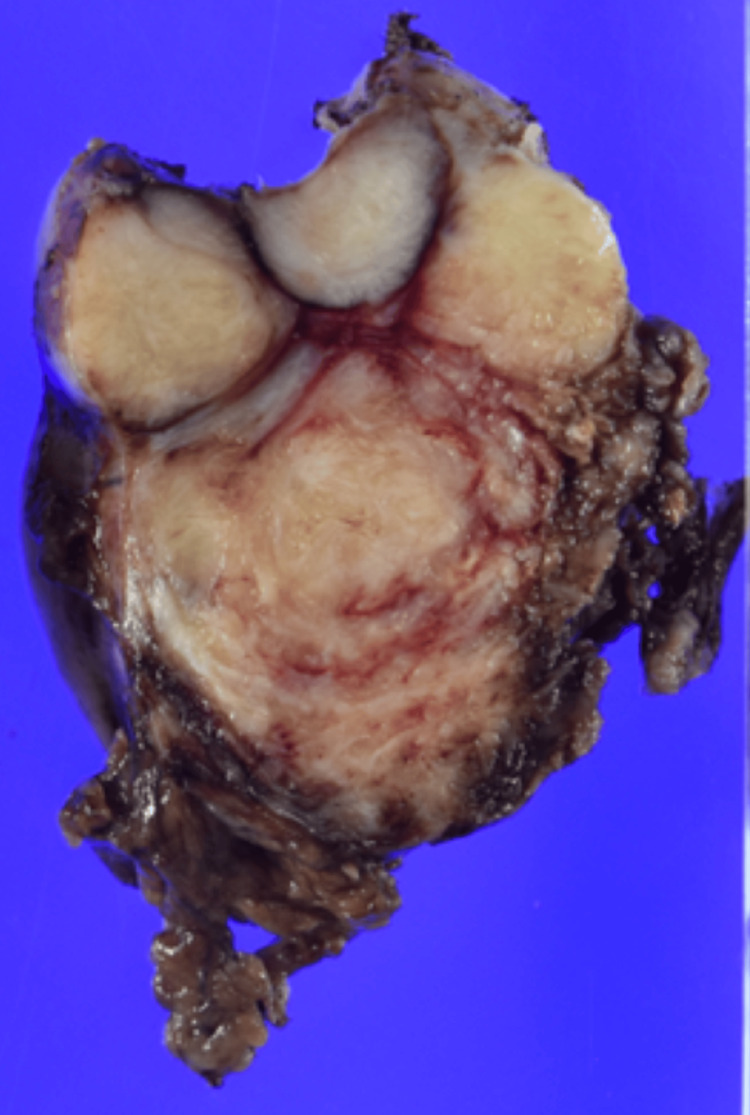
Postoperative pathology: macroscopic solitary fibrous tumor The tumor diameter was 6 × 5.5 × 4 cm. The proliferation of spindle-shaped cells showing mild-to-moderate atypia

**Figure 11:**
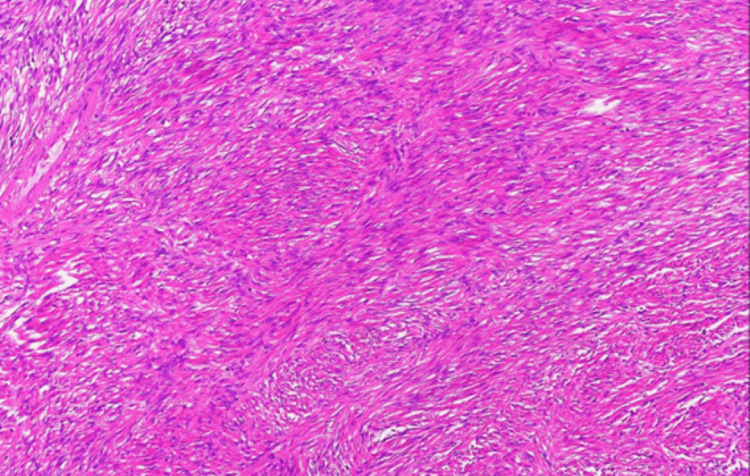
Postoperative pathology: microscopic solitary fibrous tumor Calcification (-), necrosis (-), and mitotic count (0/10 HPF) HPF: high-power field

The specimen was a 6 × 5.5 × 4 cm tumor. Macroscopically, it was described as an elastic hard internal white tumor, and microscopic examination revealed a disorganized proliferation of mild-to-moderate spindle-shaped cells. Nuclear pleomorphism, mitosis, calcification, or necrosis was not observed (Figures 10, 11).

**Figure 12:**
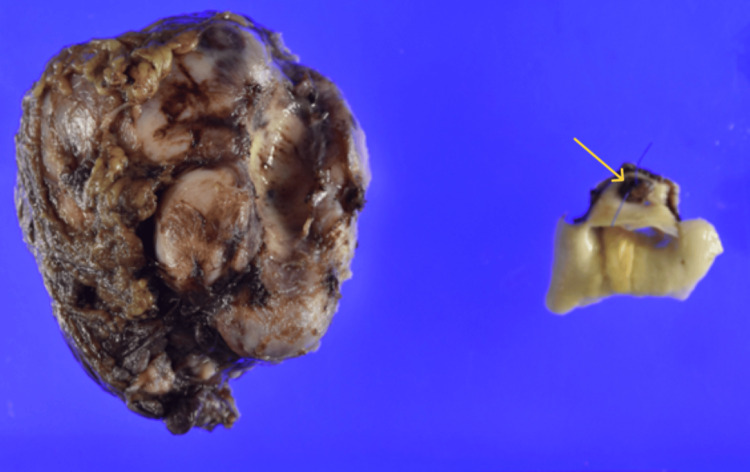
Postoperative pathology: macroscopic solitary fibrous tumor (SFT) remnant in the aortic wall Macroscopically, the SFT showed an infiltration of the aortic wall (arrow)

**Figure 13:**
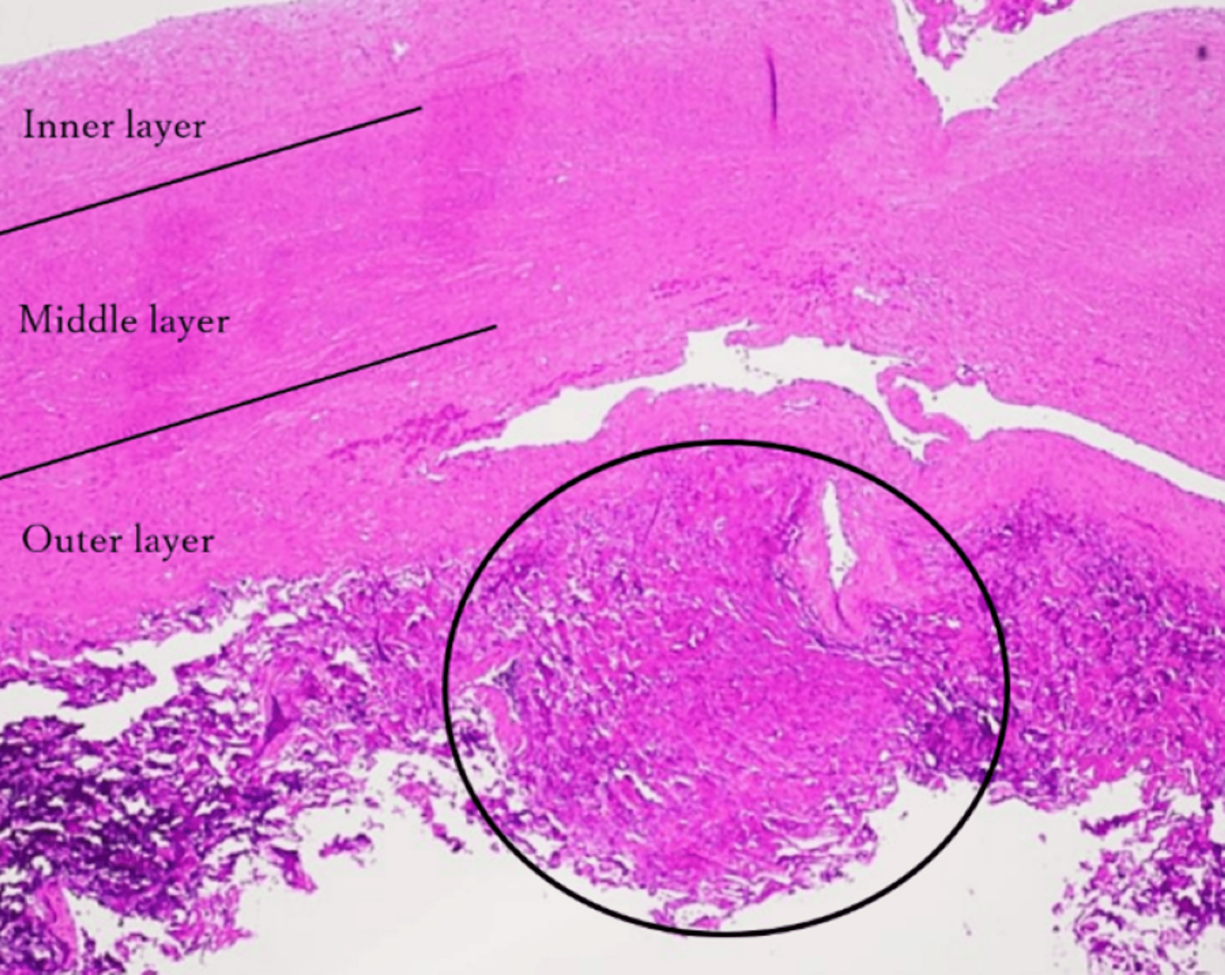
Postoperative pathology: microscopic solitary fibrous tumor (SFT) remnant in the aortic wall Microscopically, the tumor cells invaded the aortic adventitia (circle)

Tumor cells infiltrating the aortic adventitia were also observed (Figures 12, 13).

Clinical course

The patient was discharged from the hospital on postoperative day 9 without major complications and remained asymptomatic, without recurrence for nine months after surgery.

## Discussion

SFTs have a relatively good prognosis if the tumor can be completely resected, while the risk of local recurrence and distant metastasis is relatively low; however, various factors such as extrathoracic lesions, positive margins, tumor diameter of >10 cm, mitotic count of >4/10 high-power field (HPF), nuclear pleomorphism, increased cell count, and the presence of necrotic tissue increase the risk of relapse.

Demicco et al. [4] published a risk stratification model that classified distant metastasis and mortality based on risk factors such as age, tumor diameter, and mitotic count. The present case was classified as low risk with a score of 1 for a tumor diameter of 65 mm in a patient <55 years of age and a mitotic count at 0/10 HPF. Similarly, according to the risk stratification model reported by Salas et al., the patient had a score of 0, indicating a good prognosis [5]. Tolstrup et al. [6] reported that Ki-67 expression was associated with the risk of recurrence. In this case, the Ki-67 index was low.

Two points need to be discussed in this study.

The first point was the necessity of artificial vessel replacement. Georgiesh et al. [7] reported that local recurrence occurs in 19% of cases; therefore, R0 resection is preferred. In 2019, Kotani et al. [8] reported a case of an SFT that required prosthetic replacement in which the SFT was in contact with the distal aortic arch. The authors reported that the tumor invaded the aortic adventitia; therefore, artificial vessel replacement was performed to prevent recurrence from tumor residual cells. In our case, the tumor had invaded the aortic adventitia, and artificial vessel replacement was necessary to prevent relapse. Considering the patient’s age and general condition, this procedure was feasible. Our patient was relatively young, in his 40s, and the risk of recurrence or metastasis would be higher in the presence of tumor remnants; therefore, the aim was to minimize recurrence risk.

The second point was whether artificial vessel replacement could have been planned preoperatively. Since the tumor was in semi-peripheral contact with the aorta on imaging before surgery and no evidence of fatty tissue interposition was noted, it was possible that the tumor was quite close to the aorta; therefore, the possibility of partial tissue invasion was considered.

In 2002, Kume et al. [9] reported a case in which SFT arising in the retroperitoneum was in contact with the superior mesenteric artery (SMA); however, no infiltration was noted, and the SMA was dissected in clean margins. In our case, a preoperative CT-guided biopsy revealed a low-grade tumor, and no obvious aortic wall deformation was noted on imaging, which may have led to an underestimation of tumor extension.

Accurately predicting the degree of invasion preoperatively is challenging; in cases where the tumor in contact with the SMA can be dissected, however, it is suspected to be invasive. Therefore, when the tumor is in close contact with the aorta on imaging, even if the tumor is preoperatively estimated to be low grade, the possibility of invasion should be considered, and a preoperative evaluation should be performed by the vascular surgical team.

## Conclusions

SFTs have a good prognosis in the case of complete tumor excision; however, the risk of recurrence or distant metastasis varies depending on tumor size, pathological grade, age, and the presence of tumor remnants. In the case presented, although the SFT was a low-grade tumor and the patient was young, abdominal aortic replacement after complete tumor resection was performed to reduce the risk of recurrence. Additionally, when the SFT and aorta are in contact, the possibility of invasion should be considered, and a cardiovascular surgery team should perform a preoperative assessment to ascertain efficiency and safety during the procedure.

## References

[1] Klemperer P, Coleman BR (1992). Primary neoplasms of the pleura. A report of five cases. Am J Ind Med.

[2] Gold JS, Antonescu CR, Hajdu C (2002). Clinicopathologic correlates of solitary fibrous tumors. Cancer.

[3] Rajeev R, Patel M, Jayakrishnan TT, Johnston FM, Bedi M, Charlson J, Turaga KK (2015). Retroperitoneal solitary fibrous tumor: surgery as first line therapy. Clin Sarcoma Res.

[4] Demicco EG, Park MS, Araujo DM (2012). Solitary fibrous tumor: a clinicopathological study of 110 cases and proposed risk assessment model. Mod Pathol.

[5] Salas S, Resseguier N, Blay JY (2017). Prediction of local and metastatic recurrence in solitary fibrous tumor: construction of a risk calculator in a multicenter cohort from the French Sarcoma Group (FSG) database. Ann Oncol.

[6] Tolstrup J, Loya A, Aggerholm-Pedersen N, Preisler L, Penninga L (2024). Risk factors for recurrent disease after resection of solitary fibrous tumor: a systematic review. Front Surg.

[7] Georgiesh T, Aggerholm-Pedersen N, Schöffski P (2022). Validation of a novel risk score to predict early and late recurrence in solitary fibrous tumour. Br J Cancer.

[8] Kotani S, Inoue Y, Haida H, Dehari R, Kato I (2019). A rare solitary fibrous tumor originating from the distal aortic arch. Ann Thorac Surg.

[9] Kume M, Komori K, Inoguchi H, Shoji T, Furuyama T, Sakamoto A, Sugimachi K (2002). Solitary fibrous tumor in the retroperitoneal space: report of a case. Surg Today.

